# The Evolution of Artificial Intelligence in Biomedicine: Bibliometric Analysis

**DOI:** 10.2196/45770

**Published:** 2023-12-19

**Authors:** Jiasheng Gu, Chongyang Gao, Lili Wang

**Affiliations:** 1 Department of Computer Science University of Southern California Los Angeles, CA United States; 2 Department of Computer Science Northwestern University Evanston, IL United States; 3 Department of Computer Science Dartmouth College Hanover, NH United States

**Keywords:** bibliometrics, trend forecasting, AI in medicine, Word2Vec, regression models, agglomerative clustering, usage, artificial intelligence, utilization, biomedical, effectiveness, AI trends, predictive model, development

## Abstract

**Background:**

The utilization of artificial intelligence (AI) technologies in the biomedical field has attracted increasing attention in recent decades. Studying how past AI technologies have found their way into medicine over time can help to predict which current (and future) AI technologies have the potential to be utilized in medicine in the coming years, thereby providing a helpful reference for future research directions.

**Objective:**

The aim of this study was to predict the future trend of AI technologies used in different biomedical domains based on past trends of related technologies and biomedical domains.

**Methods:**

We collected a large corpus of articles from the PubMed database pertaining to the intersection of AI and biomedicine. Initially, we attempted to use regression on the extracted keywords alone; however, we found that this approach did not provide sufficient information. Therefore, we propose a method called “background-enhanced prediction” to expand the knowledge utilized by the regression algorithm by incorporating both the keywords and their surrounding context. This method of data construction resulted in improved performance across the six regression models evaluated. Our findings were confirmed through experiments on recurrent prediction and forecasting.

**Results:**

In our analysis using background information for prediction, we found that a window size of 3 yielded the best results, outperforming the use of keywords alone. Furthermore, utilizing data only prior to 2017, our regression projections for the period of 2017-2021 exhibited a high coefficient of determination (R^2^), which reached up to 0.78, demonstrating the effectiveness of our method in predicting long-term trends. Based on the prediction, studies related to proteins and tumors will be pushed out of the top 20 and become replaced by early diagnostics, tomography, and other detection technologies. These are certain areas that are well-suited to incorporate AI technology. Deep learning, machine learning, and neural networks continue to be the dominant AI technologies in biomedical applications. Generative adversarial networks represent an emerging technology with a strong growth trend.

**Conclusions:**

In this study, we explored AI trends in the biomedical field and developed a predictive model to forecast future trends. Our findings were confirmed through experiments on current trends.

## Introduction

### Artificial Intelligence in Biomedicine

Medicine has long been recognized as a prime area for applying artificial intelligence (AI) [1], with biomedicine being a vibrant and promising field. Advances in technology and science have led to the use of various methods to obtain biomedical data, such as clinical analyses, biological parameters, and medical imaging. However, the diversity and complexity of these data, along with the need for more information on certain atypical diseases result in unbalanced and nonsmooth biomedical data. In this scenario, machine learning can improve medical big data analysis, reduce the risk of medical errors, and generate a more unified diagnostic and prognostic protocol.

Recent AI research has leveraged machine learning methods to identify patterns and complex interactions from data, which require large amounts of data as support. Artificial neural networks and deep learning are currently among the most popular machine learning technologies. These methods are used in biomedicine across all medical dimensions, from genomic applications such as gene expression to public health care management such as for predicting population information or infectious disease outbreaks [2]. AI has also significantly impacted biomedical processors such as electrocardiogram, electroencephalogram, and electromyography classification processors and hearing aid processors [3].

AI is increasingly being utilized in a variety of applications in the biomedical field. Notable examples include IBM Watson-Oncology, which selects drugs for cancer treatment with equal or superior efficiency compared to human experts; Microsoft’s Hanover project at Oregon, which personalizes cancer treatment plans through analysis of medical research; and the UK National Health Service utilizing Google’s DeepMind platform to detect health risks by analyzing mobile app data and medical images from patients. Additionally, algorithms developed at Stanford University have been shown to detect pneumonia more accurately than human radiologists; in the diabetic retinopathy challenge, the computer was as effective as an ophthalmologist in making referral decisions [4]. Therefore, it is essential to analyze the trends in the integration of these AI-related technologies with the biomedical field to understand which technologies have played an important role in the past, predict the current and emerging technologies that are more likely to be important in the future, and determine which original technologies are regaining importance in a particular biomedical field.

Language models offer an effective means to analyze texts and have become the basis for many applications, including machine translation and text classification. In all text-related fields, language models can bring new improvements and opportunities to a greater or lesser extent and assist in literature research.

### Co-word Analysis

Recently, increased attention has been paid to the management of references and expansion of the research scope. Bibliometric analysis summarizes the structure of a field by analyzing the social and structural relationships between different research components such as authors, countries, institutions, and topics. Additionally, bibliometric analysis significantly impacts reorienting research and identifying popular issues. Thus, bibliometric analysis enables discovery of how research in a given field is distributed and changing. The data collected and the conclusions drawn from a bibliometric analysis can be used to track popular topics, predict promising technologies, and assist scientists in redirecting their research. There has been substantial research and application of bibliometric analysis in academia and industry, and extracting keywords to analyze texts is a very common strategy in such studies. Although it is intuitive to use the whole text as an object of analysis, this requires extensive computational resources. Moreover, many texts are not of high quality, some of them are repetitive or have no actual content, and a lot of noise can make the model learn the wrong knowledge. Therefore, keyword-focused analysis is often a better choice. Co-word analysis is one such technique that focuses on keywords and analyzes the content itself [5]. This analysis aims to uncover the intrinsic connections of articles and discover trends within them with applications in many fields, including medicine and business.

Co-word analysis was first proposed by French bibliometricians in the late 1970s [6] as a technique for studying keywords in the content of publications. Words in the co-word analysis are typically derived from the article title, abstract, and full text. These words may be specifically extracted from certain parts of each component, depending on the goal of the analysis. Co-word analysis assumes that words that frequently occur together have thematic relationships with each other. Based on this assumption, co-word analysis can be used to predict future research in a field. Analysis of the keywords of published articles in a given field has the potential to predict keywords for future research in the field, which in turn portrays the future of the research field accordingly. Co-word analysis uses several methods based on covariate matrices, such as factor, cluster, multivariate, and social network analyses. These methods help researchers to obtain an overview of a field. Thus, co-word analysis is a method to analyze papers in a field and make valid judgments.

### Text Similarity

Text similarity measurement is fundamental to natural language processing tasks and is essential in information retrieval, question answering, machine translation, and dialogue systems, among other applications. In recent years, various techniques for measuring semantic similarity have been proposed. Text similarity techniques can be divided into two main categories: text distance and text representation [7].

Text distance describes the semantic similarity of two text words from the perspective of distance. Length-based and distribution-based distance are the two main types of text distance. Traditionally, text similarity is evaluated by measuring the length distance, which uses the numerical properties of the text to calculate the text vector distance length, such as the Euclidean distance, cosine distance, or Manhattan distance [8]. However, the text similarity should not be symmetric and the length distance does not consider the statistical characteristics of the data. The distribution distance determines the similarity between documents based on the similarity of their distribution, such as Jensen-Shannon divergence [9], Kullback-Leibler divergence [10], and Wasserstein distance [11], among others.

Text representation methods convert text to a numerical feature vector. These methods are mainly divided into a string-based method, corpus-based method, semantic text matching, and graph structure–based method. String-based methods operate on string sequences and character compositions to measure the similarity or dissimilarity (distance) between two text strings for approximate string matching or comparison. The advantage of such methods is that they are simple to compute. Representative string-based methods include longest common subsequence [12], Edit distance [13], Jaro similarity [14], Dice [15], and Jaccard [16]. The corpus-based methods use information from the corpus to compute text similarity; this information can be either text features or co-occurrence probabilities. In recent studies, corpus-based approaches include three different measures: the bag-of-words model, distributed representation, and matrix decomposition method. The corpus-based methods mainly include bag-of-words [17], text frequency-inverse document frequency [18], Word2Vec [19], latent semantic analysis [20], and others. Semantic similarity determines the similarity between text and documents based on their meaning rather than character-by-character matching. Deep-structured semantic models [21] are typical models in this regard. Graph-based text similarities are mainly based on a knowledge-graph representation and a graph neural network representation. The graph structure better enables determining the similarity between nodes. Knowledge graphs [22] and graph neural networks [23] are the main methods for exploiting a graph structure.

### Predicting the Future of AI in Health Care

Some previous works have also discussed the application of AI in medicine and possible future directions. One is integrative analysis [24], where data from different modalities can describe various aspects of a health problem. By mining these heterogeneous data in an integrated way, holistic and comprehensive insight into health can be obtained. In recent years, there has been a growing number of studies and initiatives related to AI in health, integrating different aspects of clinical data and linking drug development to clinical data. AI for precision medicine [25] represents another promising combination of AI and medicine, which assists in solving the most complex problems in personalized care. For example, AI in microscopic diagnostics [26] can improve the work of pathologists and may even gradually replace their work.

In this study, we used language models to measure the relationship between keywords, which can subsequently assist in building aggregation models and using adjacent keywords. Specifically, we propose a background-enhanced prediction method for constructing data for prediction using adjacent keywords, which refer to matrices adjacent to a 2D correlation matrix constructed using a clustering algorithm. This approach allows regression models to learn better and more accurately predict the relationships between keywords. We applied this approach to predict the future trend of AI technologies used in different biomedical domains based on past trends of related technologies and biomedical domains. We further compared the prediction results to the patterns of current trends to evaluate the reliability of the prediction.

## Methods

### Data Sets

The data sets used in this study were obtained from the National Institutes of Health PubMed and PMC collections, with measures taken to avoid duplication by utilizing unique identifiers.

The corpus utilized in this study consists of three parts: (1) 114,266 abstracts and 49,126 full texts from PubMed and PMC obtained by searching keywords such as “machine learning,” “data mining,” “artificial intelligence,” “deep learning,” and “classifier” in the Title/Abstract field; (2) 61,382 full-text papers from PMC obtained by searching keywords such as “machine learning,” “data mining,” “artificial intelligence,” and “deep learning” in all fields, serving as a complement to the previous part; and (3) 2,507,391 full-text papers retrieved from the PubMed Central Open Access section with no keyword filtering to capture a comprehensive understanding of the biomedical field.

Due to permission restrictions, full-text access was limited for some papers. The full text of the papers primarily served for training, with the core of our experiments lying in the analysis and model prediction based on the abstracts.

### Language Model

We utilized the word-embedding model Word2Vec as our language model owing to its advantages of efficiency and robustness among other available options [27].

Word embedding is a method of transforming a single word into a digital representation that captures various features of the word within a text, such as semantic relationships, definitions, and contexts. These digital representations can be used to identify similarities or dissimilarities between words.

To feed text data into a machine learning model, the text must be converted into an embedding. A simple method to achieve this involves “hot-coding” the text data, where each vector is mapped to a category. However, such simple embeddings have limitations as they do not capture the features of the words and can be large depending on the corpus size.

The effectiveness of Word2Vec is derived from its ability to combine vectors of similar words, leading to reliable estimates of word meaning based on their frequency in the corpus. This results in associations with other words, such as similar embedding vectors of “king” and “queen.” Algebraic operations on word embeddings can also provide approximations of word similarity, such as obtaining the vector for “queen” by subtracting the vector for “man” from the vector for “king” and adding the vector for “woman.” The cosine similarity measure is used to compare the similarity of two words, which is calculated according to the following formula:

*cos*(*x*,*y*)=*x*·*y*/∣*x*∣×∣*y*∣

To improve the suitability of the original corpus for our language model, we performed extensive preprocessing to address any noise that may impact the model’s effectiveness. This included removing all numeric and nonalphabetic characters, except for the special character “-,” which is often used to link multiple words and create unique phrases. Additionally, to enhance the word vectors of biomedical- and AI-related keywords, we transformed multiword keywords in 114,266 abstracts into single tokens by merging them; for example, “machine learning” was merged into “machine+learning.”

The selection of hyperparameters was based on the available computational resources and the training corpus size. Our Word2Vec model had 300 dimensions and a window size of 5. Our computational device is a cluster with 384 GB of memory and 16 CPU cores. The Word2Vec model was trained sequentially on the three data sets, with the entire training process taking approximately 72 hours.

### Background-Enhanced Prediction

Technology tends to be heavily studied in similar areas of research. Conversely, technology and its similar variants may be very popular in the same field. For example, techniques used for one type of cancer may also be relevant to other types of cancer, and various artificial neural models can all be applied in the field of medical image recognition. Our model was developed to predict future research trends based on direct relationships between technologies and fields and related technologies and fields. More specifically, we extracted the top 500 most frequent AI terms and the top 1000 most frequent biomedical fields from the 114,266 abstracts. To distinguish AI terms from biomedical terms, we adopted a simple classifier. We obtained approximately 47,000 biomedical phrases from Medical Subject Headings and approximately 700 AI algorithms from Wikipedia. We used the average cosine similarity of each keyword and all terms in the two-word sets to predict whether the keyword should belong to the biomedical or AI domain. Next, Word2vec was used to obtain embeddings from each word. After converting all words into embeddings using Word2vec, we applied agglomerative clustering [28] to classify all the keywords according to their embeddings. Agglomerative clustering is a bottom-up clustering process. Initially, each input object forms its cluster. In each subsequent step, the two “closest” clusters are merged until only one remains. In our case, words with similar meanings will be grouped. Such a hierarchy is useful in many applications, and we provide the resulting tree diagram next to the corresponding heat map to best visualize the relationships between the surrounding categories.

**Figure 1:**
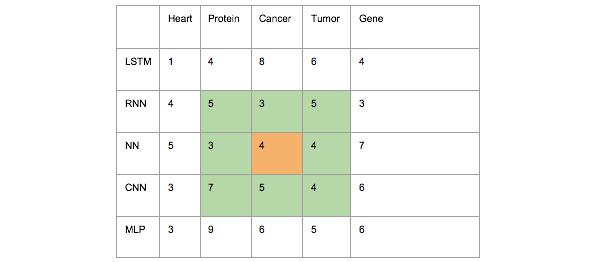
Co-occurrence frequency table of biomedical- and artificial intelligence–related keywords. Each number represents the number of co-occurrences of a given artificial intelligence model and biomedical term. The orange part represents the information held by the keyword and the green part represents the information held by the keyword's neighbors. CNN, convolutional neural network; LSTM: long short-term memory; MLP, multilayer perceptron; NN: neural network; RNN, recurrent neural network.

Figure 1 depicts the co-occurrence frequency of biomedical and AI keywords. For regression prediction, we utilized not only the data from the orange part (information held by the keyword) but also from the green part (information held by the word neighboring the keyword). This inclusion provides a richer context, offering models that include more relevant information to learn from. The number 4 in the orange cell indicates the number of co-occurrences of “neural network” and “cancer,” which we not only used as input to predict the number of future co-occurrences of the terms “neural network” and “cancer” but also added the number of co-occurrences in the green section, 5+3+5+3+4+7+5+4, to obtain a more comprehensive prediction using the neighboring information.

### Regression Model

The inputs and outputs of the regression model represent the co-occurrence frequency of biomedical and AI keywords in previous years and the co-occurrence frequency of future biomedical and AI keywords obtained by prediction. Due to the limited number of AI-related papers from 1970 to 2000, we used semiannual statistics for January 2000 to December 2021 in our analysis. We incorporated each semiannual data set into a training and testing prediction model. Our model uses a small window of the heatmap for the past 6 months, which was constructed using specific technology and domain pairs as features, and the model was trained on all prior-year samples to predict the current year’s heat level. We employed six different regression algorithms: support vector regression, lasso regression, ridge regression, elastic net [29], orthogonal matching pursuit [30], and passive aggressive regressor [31], using Scikit-learn [32]. We set the parameters to random_state=0 for lasso, ridge, elastic net, and passive aggressive regressor; normalize=True for lasso and ridge; and left the other parameters as default values.

The data from 2016 to 2021 were used as a validation set and the data from 2002 to 2021 were used to predict trends from 2021 to 2026.

## Results

### Visualization

**Figure 2:**
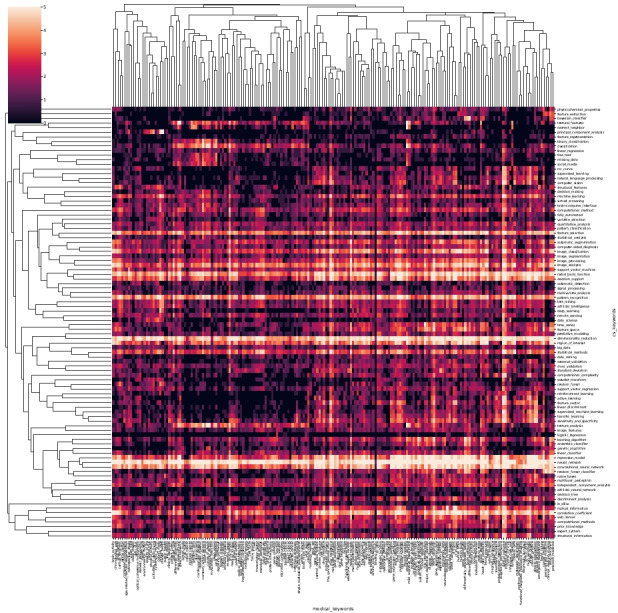
Heatmap of the publications related to certain artificial intelligence (AI) technologies and biomedical fields from 1970 to 2021. The horizontal axis is the keywords in the biomedical field and the vertical axis is the keywords of AI technology. A higher resolution version of this figure is available in Multimedia Appendix 1.

Figure 2 presents a heatmap that illustrates the distribution of publications from 1970 to 2021. To improve the visualization, we limited the analysis to the top 100 frequently occurring AI terms and the top 200 frequently occurring biomedical terms. However, in subsequent experiments, we expanded the analysis to include the top 500 AI terms and the top 1000 biomedical terms. The heatmap plots the intersection of computer technology and biomedical fields, with the heat representing the logarithm of the number of papers published between 1970 and 2021 that mention both areas in the abstract. This map demonstrates that neural network–based methods are the most popular AI tools for application in the medical field.

**Figure 3:**
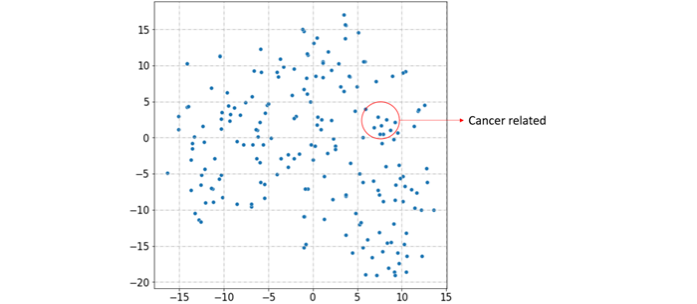
Biomedical keywords in a t-distributed stochastic neighbor embedding plot.

**Figure 4:**
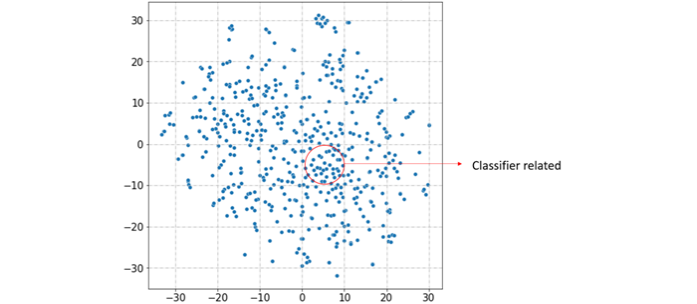
Artificial intelligence keywords in the t-distributed stochastic neighbor embedding plot.

After encoding words using Word2Vec, each word becomes a corresponding embedding. To evaluate the quality of the generated embeddings, we employed t-distributed stochastic neighbor embedding (t-SNE) [33], a technique for visualizing high-dimensional data by projecting it onto a 2D map. The t-SNE plots in Figures 3 and 4 reveal that the word embeddings obtained by Word2Vec do allow words with similar meanings to be close together in the embedding space. Figure 3 highlights the vector positions of cancer-related keywords in 2D space, while Figure 4 shows the positions of classifier-related keywords.

### Future Trend Prediction

**Figure 5:**
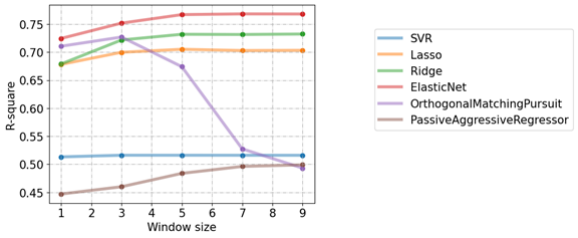
Mean R-square values obtained by forecasting in half-yearly intervals from July 2002 to December 2021 under different window sizes for different methods. SVR: support vector regression.

Figure 5 illustrates the average R^2^ values of all predicted and actual results from July 2002 to December 2021, with different window sizes of 1, 3, 5, 7, and 9. From Figure 5, we can also see that the elastic net model provided the best results when the window size was equal to 9, whereas some other models worked best when the window size was equal to 3. 

**Figure 6:**
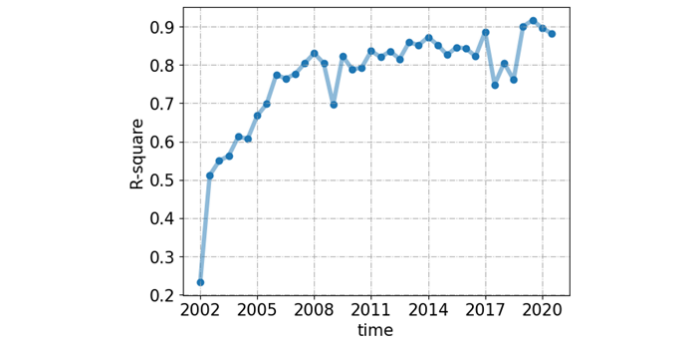
Line graph of the forecast results for each half year from 2002 to 2021. The model used was elastic net with a 9×9 window size, as this resulted in the best prediction (R-square value).

**Figure 7:**
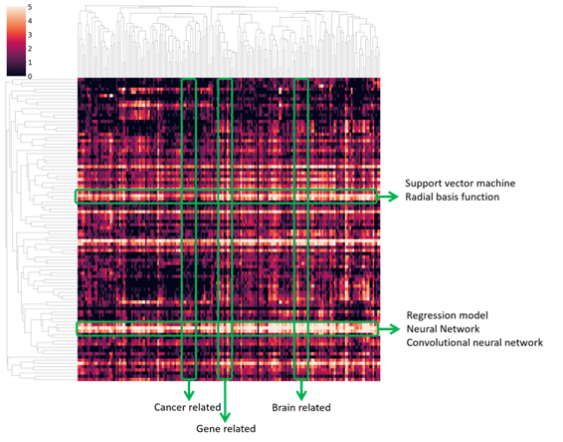
The predictions are iterated in half-year increments from July 2014 to December 2021, and the data obtained from the predictions are used as the data set for the subsequent prediction models for training. The horizontal axis is time and the vertical axis is the R-square value. A higher resolution version of this figure is available in Multimedia Appendix 2.

**Figure 8:**
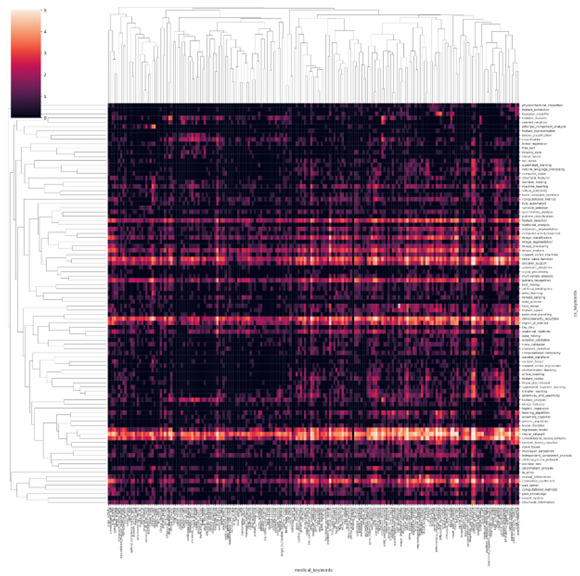
Heatmap from July to December 2021 for the actual intersection of artificial intelligence (AI) technology and biomedical field applications. The horizontal axis is the keywords in the medical field and the vertical axis is the keywords in AI technology. A higher resolution version of this figure is available in Multimedia Appendix 3.

**Figure 9:**
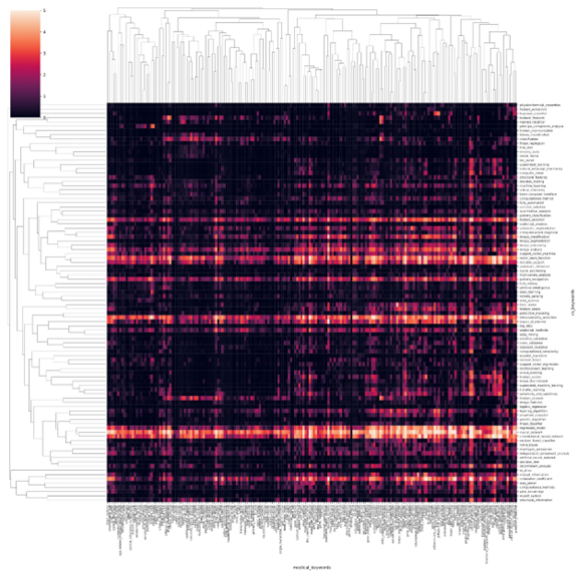
Predicted heat map of the intersection of artificial intelligence (AI) technology and biomedical field applications from July to December 2021. The horizontal axis is the keywords in the medical field and the vertical axis is the keywords in AI technology. A higher resolution version of this figure is available in Multimedia Appendix 4.

**Figure 10:**
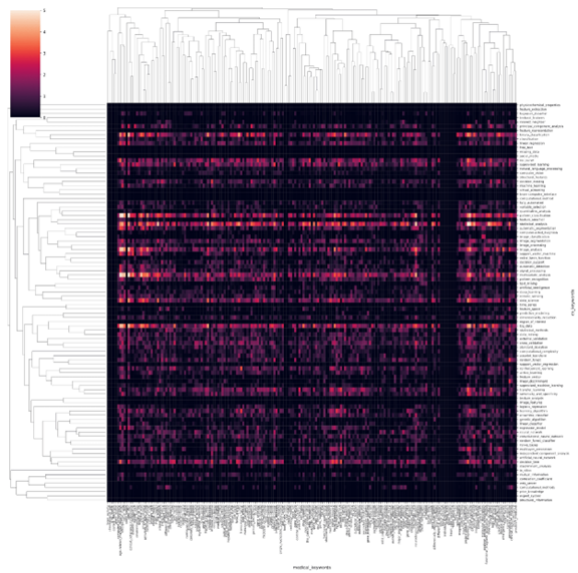
Heatmap drawn from the difference between the predicted and actual heatmaps for July to December 2021 (Figures 9 and 8, respectively) representing the intersection of artificial intelligence (AI) technology and biomedical field applications. The horizontal axis is the keywords in the medical field and the vertical axis is the keywords in AI technology. A higher resolution version of this figure is available in Multimedia Appendix 5.

Since our model relies on the previous year’s heatmap as a feature, to predict a longer time horizon, we iteratively ran our model using the predicted heatmap of cycle x to predict the heatmap of cycle x+1. As shown in Figure 6, although the R^2^ value decreased during the 5-year prediction, it was still relatively high. We also provide a 100×200 demonstration to visualize the prediction results in Figures 7-10. These heatmaps, like those in Figure 2, are also used to show the frequency of co-occurrence between the keywords of AI technology and biomedicine. Figure 8 depicts the original publications that were recorded between July and December 2021, while Figure 9 represents the predicted publications for the same time period. To effectively showcase the disparity between the actual and projected outcomes, a heatmap was generated using both the original and predicted heatmaps. This comparison is visually presented in Figure 10, allowing for a clear and easily understandable differentiation between the two sets of data.

### Co-occurrence Trend Analysis

The data obtained through statistical analysis indicated that the number of papers combining AI with biomedicine is increasing in spurts. From Table 1, we can see which combinations between AI and biomedicine are the most popular. The field of genetics shows many combinations with various AI technologies, occupying 13 of the top 20 positions. Numerous papers on this topic highlight its popularity [34-36]. The combination of AI and protein ranked fourth, demonstrating that protein analysis is a very suitable field for the use of machines. Cancer and tumors are currently the main challenges in biomedicine, and their combination with AI is also a popular topic at present. In these biomedical fields, machine learning is the AI technology with the highest number of applications. Although deep learning and neural networks are trendy, traditional methods such as vector automata and random forests are still the main choices in biomedical fields. Many fundamental concepts of AI are also included in this ranking, such as classification, regression, cross-validation, feature extraction, receiver operating characteristic, and others. Overall, this analysis shows that AI has become a key technology in the biomedical field and requires the proficiency of biomedical scientists.

**Table 1:** The top 20 combinations of artificial intelligence (AI) technologies and biomedical fields that have appeared in the literature in the last 5 years.

Rank	AI technology, biomedical field	Proportion of publications, %
1	machine learning, gene	1.650
2	classification, gene	1.038
3	neural network, gene	0.634
4	deep learning, gene	0.453
5	support vector machine, gene	0.447
6	machine learning, protein	0.404
7	regression, gene	0.402
8	learning algorithm, gene	0.385
9	machine learning, cancer	0.380
10	classification, cancer	0.351
11	random forest, gene	0.331
12	artificial intelligence, gene	0.249
13	convolution neural network, gene	0.241
14	cross-validation, gene	0.219
15	feature selection, gene	0.191
16	neural network, cancer	0.176
17	classification, tumor	0.172
18	supervised learning, gene	0.171
19	machine learning, tumor	0.171
20	receiver operating characteristic, gene	0.169

Since many combinations between AI and biomedicine have a very small contribution or are nonexistent, some are not meaningful; therefore, we set a reasonable threshold to filter such combinations, avoiding the situation where the original minimal combination grows by a considerable percentage with little growth so that the table showing the trend of changes is more meaningful. From Table 2, we can see the very rapid growth of cases combining AI and biomedicine in the last 5 years. This is because genes, proteins, oncology, and many other fields are growing rapidly, and core medical testing technology such as magnetic resonance imaging is compatible with AI.

We used the best model from our proposed methodology to forecast the trends in AI technology and biomedicine over the next 5 years. The prediction results for the contributions of each combination and their growth are shown in Table 3 and Table 4, respectively. The regression results were rounded for brevity of presentation in the tables. We can use these predicted results to provide an outlook on the future development of AI in biomedicine. From the point of view of AI technologies, standard techniques such as deep learning, machine learning, and neural networks still dominate. Traditional machine learning methods such as random forest and support vector machine are outside the top 20 prediction results. Deep learning will gradually become the mainstream AI technology combined with biomedicine [37]. From a biomedical perspective, genetics will continue to dominate. At the same time, studies focusing on proteins and tumors will leave the top 20 and be replaced by early diagnostics, tomography, and other detection technologies. These are certain areas that are well suited to incorporate AI technology.

**Table 2:** The 20 most rapidly growing combinations of artificial intelligence (AI) technologies and biomedical fields in the last 5 years.

Rank	AI technology, biomedical field	Growth, %
1	electronic, health records	1054.545
2	electronic health records, electronic health	1054.545
3	machine learning, electronic health record	1033.333
4	machine learning, health care	820.000
5	machine learning, risk factor	816.667
6	machine learning, public health	735.000
7	neural network, gene	700.483
8	neural network, cancer	647.059
9	machine learning, tumor	619.697
10	image analysis, gene	613.333
11	machine learning, clinical trial	572.414
12	machine learning, clinical practice	566.667
13	decision making, gene	547.619
14	artificial intelligence, gene	511.111
15	random forest, cancer	493.617
16	machine learning, clinical data	487.179
17	electronic medical record, medical records	480.000
18	next generation sequencing, gene	467.647
19	random forest, tumor	466.667
20	machine learning, magnetic resonance	456.579

**Table 3:** The top 20 combinations of artificial intelligence (AI) technologies and biomedical fields that will emerge in the next 5 years.

Rank	AI technology, biomedical field	Predicted proportion of publications, %
1	machine learning, gene	2.331
2	artificial intelligence, early diagnosis	2.289
3	artificial intelligence, early detection	1.901
4	artificial intelligence, gene	1.487
5	neural network, gene	1.392
6	deep learning, computed tomography	1.288
7	artificial intelligence, systematic reviews	1.239
8	classification, gene	1.197
9	supervised learning, gene	1.188
10	generative adversarial network, gene	1.040
11	artificial intelligence, personalized treatment	0.881
12	machine learning, risk factors	0.659
13	deep learning, gene	0.633
14	artificial intelligence, systematic review	0.617
15	convolution neural network, gene	0.604
16	learning algorithm, gene	0.593
17	receiver operating characteristic, computed tomography scans	0.581
18	machine learning, medical records	0.578
19	machine learning, blood pressure	0.569
20	artificial intelligence, imaging modalities	0.554

**Table 4:** The top 20 rapidly growing combinations of artificial intelligence (AI) technology and biomedical fields in the next 5 years.

Rank	AI technology, biomedical field	Predicted growth, %
1	artificial intelligence, gene	2253.521
2	machine learning, risk factor	2184.491
3	cross-validation, gene	2164.150
4	receiver operating characteristic, gene	1504.581
5	learning algorithm, gene	1421.751
6	neural network, gene	1340.880
7	convolution neural network, gene	1296.067
8	classification, gene	1280.985
9	machine learning, gene	1261.342
10	classification, cancer	888.106
11	support vector machine, gene	791.807
12	neural network, cancer	665.430
13	artificial intelligence, cancer	621.627
14	deep learning, gene	502.318
15	classification, tumor	415.298
16	regression, gene	377.864
17	machine learning, protein	333.778
18	random forest, gene	322.787
19	deep learning, cancer	200.080
20	natural language processing, natural language	192.518

## Discussion

### Principal Findings

#### AI Technology Trends in Biomedicine

Our findings confirm that standard AI techniques, including deep learning, machine learning, and neural networks, continue to be the primary driving forces behind the integration of AI into biomedicine. However, it is noteworthy that generative adversarial networks (GANs) [38] are gaining prominence, particularly in the genetics field. GANs hold immense potential for applications in medical imaging and drug discovery owing to their ability to generate synthetic images across various modalities.

#### Evolution of Biomedical Research

The data also highlight the shifting landscape of biomedical research. While genetics remains dominant, areas such as proteins and tumors are gradually giving way to early diagnostics, tomography, and other detection technologies. These developments align with the suitability of these fields for AI integration, resulting in promising advancements in health care analysis and diagnostics.

#### Impact of AI on Health Care

As suggested by previous research [24], the future of AI in health care is promising. AI has the potential to enhance the accuracy of cancer diagnosis and prognosis beyond that of average statistical experts [39,40]. Furthermore, as AI technology continues to advance, it will enable the resolution of more complex and specialized health care problems, further transforming the biomedical landscape.

### Future Work

By utilizing keywords to filter medical papers that have applied AI techniques, we identified key connections and trends among them. The approach of using keywords aggregated based on text similarity performed well in the regression model. This approach is intuitive and leads to improved co-word analysis for trend prediction.

Fundamentally, incorporating peripheral information led to higher regression accuracy and more accurate predictions of future trends. Additionally, this approach also takes into account internal relationships within a class compared to previous methods. However, this also raises the question of how to best measure the degree of keyword association.

We made some simple assumptions that words with similar meanings would complement the information of the others. Specifically, considering only their own meanings tends to make the predictions one-sided, while having more reference information naturally makes the predictions more robust. This can be seen as a type of data augmentation. There are still many directions to explore regarding this approach. In future research, it may be possible to use different text similarity methods such as convolutional neural network, bidirectional encoder representations from transformers, and various regression models, where the reliability of text similarity determines whether the information obtained from the surrounding context is valid. Additionally, different time spans for the prediction can be studied. Although this study focused on AI techniques in the biomedical field, the applicability of the proposed approach extends to any study involving co-word analysis.

### Limitations

While our study provides valuable insights into the trends of AI technologies in the biomedical domain based on a comprehensive data set from PubMed, there are several limitations to consider. First, there is a limitation of the data source, since our study solely relies on PubMed as the primary source of articles, which might introduce a selection bias. There are numerous other databases and grey literature sources that were not considered, and their inclusion might have offered a more comprehensive view. Second, our study lacks external validity. Our findings, although significant in the context of our data set, require validation with real-world applications and events to check their external validity.

### Conclusions

In this study, we aimed to explore the analysis and prediction of trends at the intersection of biomedical and AI research. To accomplish this, we collected a large corpus of articles from PubMed on the intersection of AI and biomedicine. Initially, we attempted to use regression on the extracted keywords alone. However, we found that this approach was lacking in information. Therefore, we proposed a method called background-enhanced prediction to expand the knowledge utilized by the regression algorithm by incorporating both the keywords and their surrounding context. This data construction method improved the performance of our forecasting models. Our findings were validated through comparisons with current trends. In particular, the integration of electronic medical record big data with AI, laboratory data, clinical trials, and imaging diagnostic tools has emerged as a prominent approach.

## Appendix

Multimedia Appendix 1 Higher resolution version of Figure 2.

Multimedia Appendix 2 Higher resolution version of Figure 7.

Multimedia Appendix 3 Higher resolution version of Figure 8.

Multimedia Appendix 4 Higher resolution version of Figure 9.

Multimedia Appendix 5 Higher resolution version of Figure 10.

## Abbreviations

AI: artificial intelligence
GAN: generative adversarial network
t-SNE: t-distributed stochastic neighbor embedding

## Data Availability

The code is available at GitHub [41].
