# Supplementary figures and images for: The Evolution of Artificial Intelligence in Biomedicine: Bibliometric Analysis
Source: JMIR AI. 2023 Dec 19;2:e45770. doi: 10.2196/45770 (PMC11041403; doi:10.2196/45770)

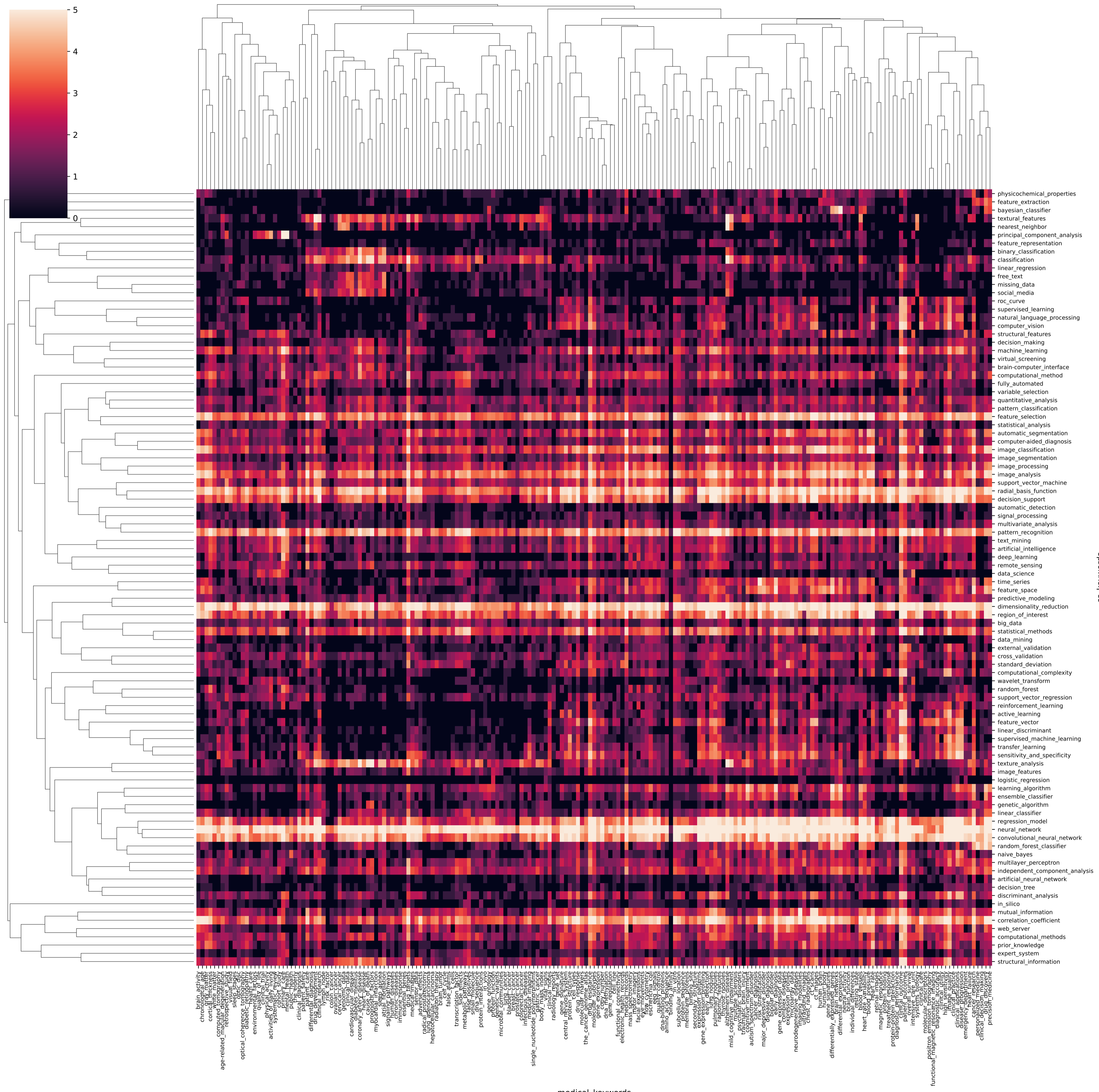

Supplement: Multimedia Appendix 1 [file ai_v2i1e45770_app1.pdf]

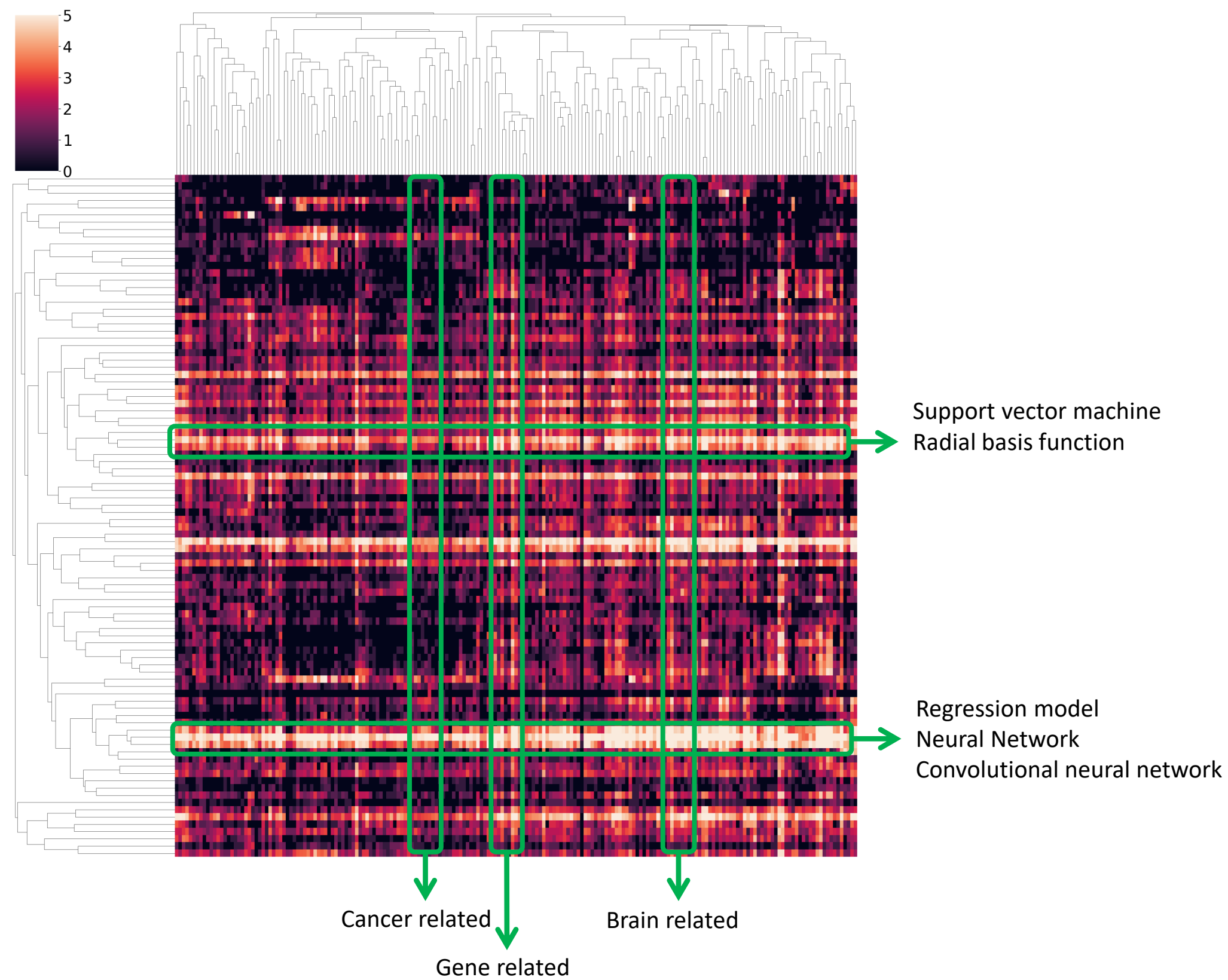

Supplement: Multimedia Appendix 2 [file ai_v2i1e45770_app2.pdf]

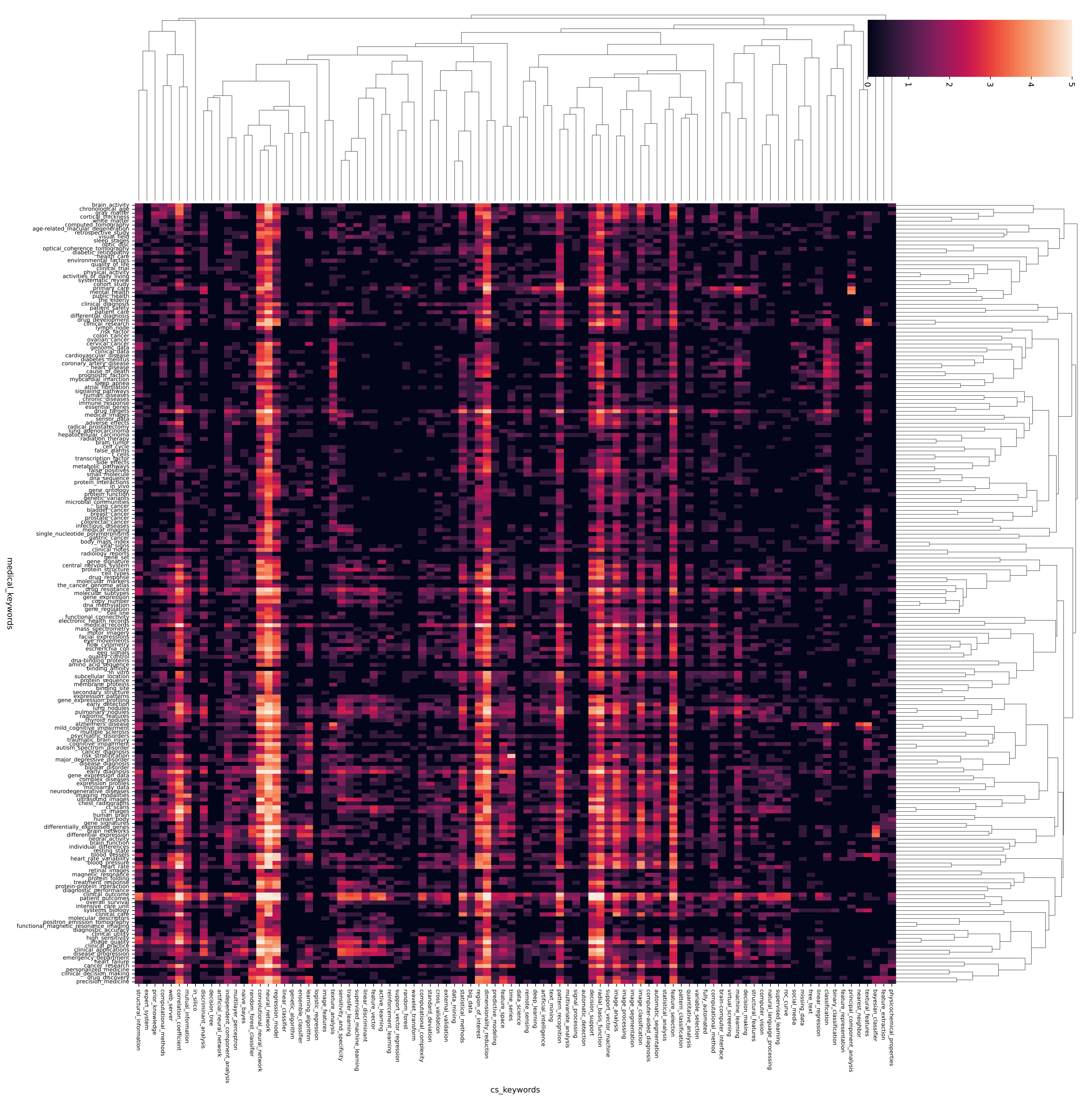

Supplement: Multimedia Appendix 3 [file ai_v2i1e45770_app3.pdf]

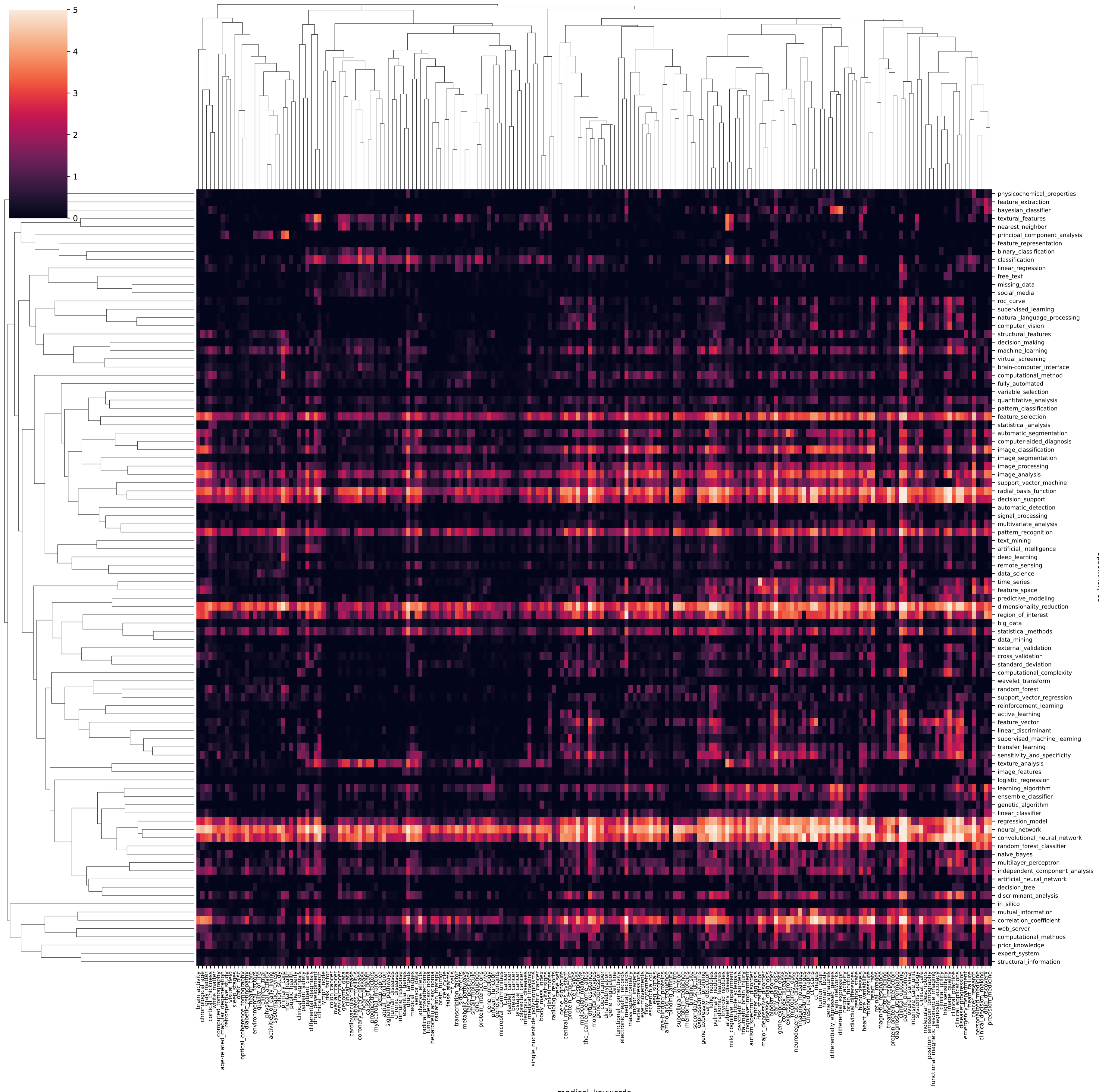

Supplement: Multimedia Appendix 4 [file ai_v2i1e45770_app4.pdf]

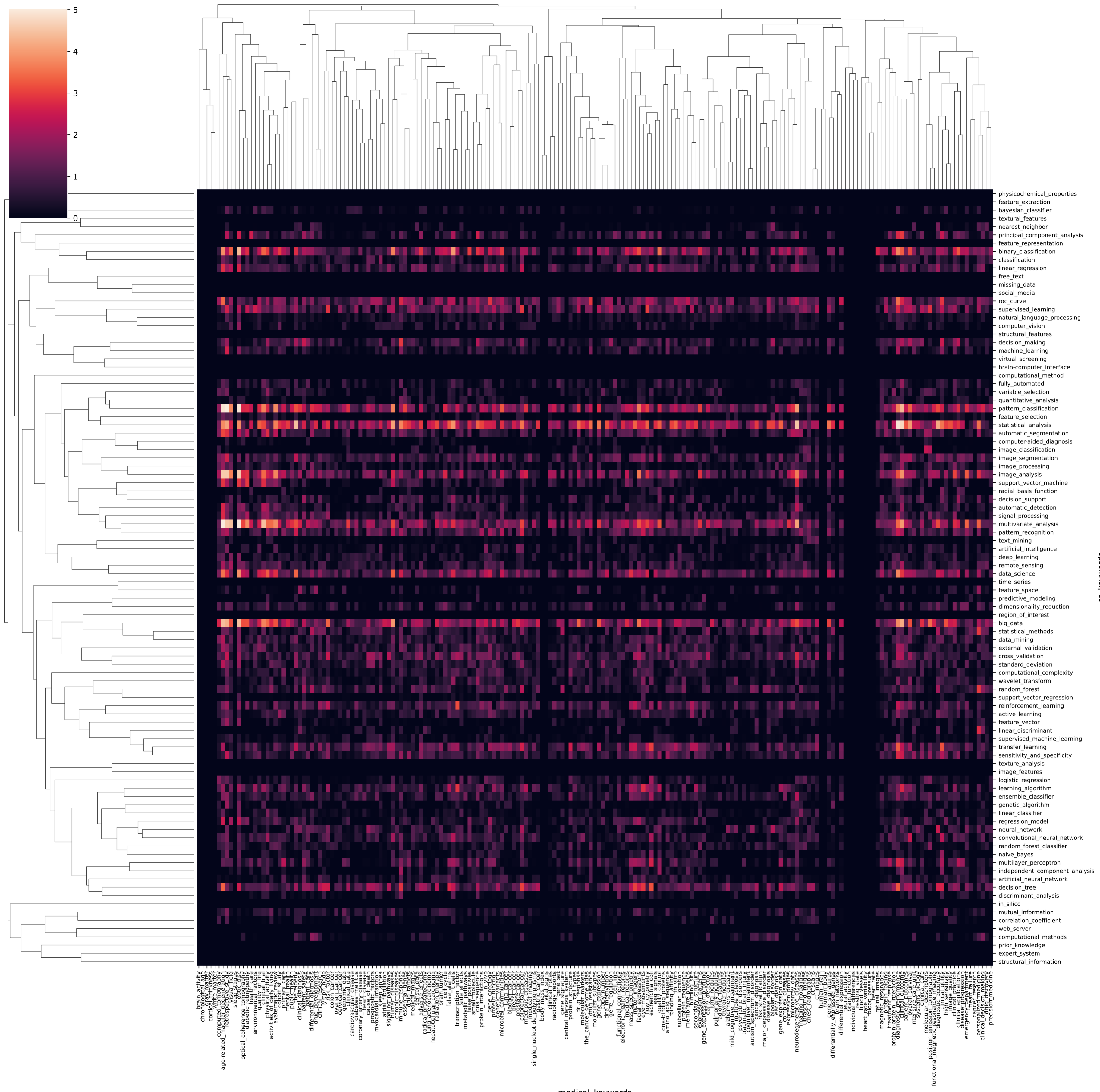

Supplement: Multimedia Appendix 5 [file ai_v2i1e45770_app5.pdf]
